# Indoor Mobility Patterns Measured by PIR Sensors for Classifying Health-Related Quality of Life in Older Adults Using Machine Learning

**DOI:** 10.3390/s26154759

**Published:** 2026-07-27

**Authors:** Diego Robles Cruz, Andrea Lira Belmar, Anthony Fleury, Méline Lam, Jean Paul Maidana, Carla Taramasco Toro

**Affiliations:** 1 Instituto de Tecnología para la Innovación en Salud y Bienestar, Facultad de Ingeniería, Universidad Andrés Bello, Viña del Mar 2520000, Chile; jean.maidana@unab.cl (J.P.M.); carla.taramasco@unab.cl (C.T.T.); 2 Departamento de Morfología, Facultad de Medicina, Universidad Andrés Bello, Santiago 8370035, Chile; a.lira@uandresbello.edu; 3 IMT Nord Europe, Institut Mines Télécom, Centre for Digital Systems, F-59653 Villeneuve d’Ascq, France; anthony.fleury@imt-nord-europe.fr (A.F.); meline.lam@etu.imt-nord-europe.fr (M.L.)

**Keywords:** passive infrared sensors, indoor mobility, digital biomarkers, aging in place, health-related quality of life, EQ-5D, machine learning

## Abstract

Passive infrared (PIR) sensors provide a low-cost, unobtrusive, and privacy-preserving approach for continuously monitoring daily activity in older adults. This study investigated whether indoor mobility features derived from PIR sensors could discriminate levels of health-related quality of life (HRQoL) in community-dwelling older adults living alone. Mobility variables were extracted from three months of PIR sensor recordings and aggregated at the participant level for 40 individuals, who were classified into high- and low-HRQoL groups according to the EQ-5D index. A nested stratified five-fold cross-validation framework was implemented, incorporating RandomOverSampler exclusively within the training folds to address class imbalance while preserving the original distribution of the outer test folds. Three machine learning classifiers—Support Vector Machine (SVM), Random Forest, and K-Nearest Neighbors (KNN)—were evaluated using accuracy, precision, recall, F1-score, and the area under the receiver operating characteristic curve (AUC). The SVM achieved the best overall performance, with an accuracy of 0.825±0.143, precision of 0.860±0.080, recall of 0.900±0.149, F1-score of 0.876±0.102, and AUC of 0.937±0.069. Random Forest achieved a comparable AUC of 0.933±0.109, whereas KNN showed lower overall performance and greater variability across the outer folds. Aggregated out-of-fold predictions further provided class-specific performance estimates while preserving the original participant distribution, confirming that model evaluation was conducted exclusively on non-oversampled test data. Overall, the findings support the feasibility of combining PIR-derived mobility features with interpretable machine learning models to investigate HRQoL in older adults. These results should be considered proof-of-feasibility and warrant validation in larger, independent, and more diverse cohorts.

## 1. Introduction

Indoor mobility is a fundamental indicator of functional status, health, and well-being in older adults. Community-dwelling seniors spend approximately 83% of their time at home [1], significantly more than middle-aged adults, who spend between 61% and 77% of their time indoors [2]. Given that such a large proportion of daily life occurs within the home environment, changes in indoor mobility patterns can provide early signals of emerging physical, cognitive, or emotional difficulties. Prior research has shown that indoor activity differs significantly between cognitively healthy older adults and those with cognitive impairment [3], and that disruptions in daily routines often constitute early manifestations of functional or cognitive decline [4]. These findings underscore the need for continuous and unobtrusive monitoring systems capable of capturing real-world patterns of daily functioning.

Building on this perspective, a growing body of evidence has strengthened the link between indoor mobility and health-related quality of life (HRQoL). Higher levels of in-home movement, reflected in metrics such as step counts, room transitions, or overall activity, are consistently associated with better self-perceived health, lower disability, and greater autonomy [5,6,7]. Conversely, reduced mobility has been linked to frailty, functional decline, and poorer quality of life, particularly among individuals living with chronic conditions [6,7]. Frailty has emerged as a key mediating factor in this relationship, whereby reduced daily mobility increases vulnerability to disability and diminished well-being. Importantly, intervention studies have demonstrated that enhancing mobility through exercise, movement stimulation, or environmental adaptations can lead to measurable improvements in quality of life and independence among older adults [8,9,10,11,12]. Collectively, these findings suggest that indoor mobility patterns constitute meaningful behavioral markers of functional health and subjective well-being.

In this context, understanding whether passively measured indoor mobility metrics are associated with HRQoL is particularly relevant for older adults living alone, who may be at increased risk of unnoticed functional decline. While movement within the home can be monitored using a variety of technologies, unobtrusive sensor-based approaches are uniquely suited for long-term deployment, as they minimize burden, preserve privacy, and enable continuous observation. Despite these advantages, relatively limited work has examined whether indoor mobility metrics can effectively discriminate levels of HRQoL, particularly when combined with supervised machine learning approaches.

Accordingly, the present study investigates whether the frequency and temporal organization of in-home mobility, recorded over a three-month period, are associated with HRQoL in older adults living alone. Participants were categorized into high and low HRQoL groups based on their EQ-5D index, and supervised machine learning models were employed to evaluate whether mobility-derived features can differentiate between these groups.

We hypothesize that:

1. Older adults with higher HRQoL will exhibit greater mobility frequency and distinct temporal mobility patterns;
2. Indoor mobility features will support the classification of HRQoL groups using supervised machine learning models.

From a technical perspective, assessing health-related quality of life through passive indoor mobility monitoring presents several challenges. These include the heterogeneity of home environments, the absence of continuous clinical labels, the need for unobtrusive and privacy-preserving sensing technologies, and the requirement for interpretable mobility metrics that remain comparable across individuals and dwellings.

Within this framework, the contribution of the present study does not lie in algorithmic novelty, but rather in the demonstration of a pragmatic and scalable approach that integrates low-cost PIR sensing, interpretable mobility-derived features, and standard machine learning models to explore associations with self-perceived quality of life in real-world home settings. By emphasizing feasibility and interpretability, this work establishes a methodological baseline for future studies incorporating richer contextual information and more advanced modeling strategies.

## 2. Previous Research and Current Study

Passive Infrared (PIR) sensors have been widely recognized as an effective, unobtrusive, and privacy-preserving technology for monitoring movement patterns within the home. Research in ambient sensing has demonstrated that activity indicators derived from PIR sensors provide reliable information about daily routines, behavioral regularity, and deviations from habitual patterns [13,14,15,16]. In addition, variability in the timing of PIR activations has been associated with gait instability and early functional decline in older adults, highlighting the potential of these sensors to capture subtle changes in functional behavior [16,17].

Building on this foundation, PIR sensors have been extensively used to characterize daily routines, detect behavioral deviations, and identify changes related to cognitive or physical decline [18,19,20]. Their clinical relevance has been demonstrated across a range of conditions, including dementia and depression monitoring [21], assessment of instrumental activities of daily living in smart home environments [22], detection of cognitive fluctuations in Alzheimer’s disease and mild cognitive impairment [23,24], and multimodal monitoring in Parkinson’s disease [25]. More recently, behavioral signatures derived from PIR sensors have also been linked to the identification of depression risk in older adults [26].

Additional studies have highlighted that PIR sensors can detect prolonged inactivity, irregular sleep and wake patterns, and subtle fluctuations in mobility that often precede clinically observable deterioration. These systems have been validated within smart home platforms supporting aging in place, offering key advantages such as low cost, feasibility for extended monitoring, and high acceptability among older adults [27]. In these contexts, PIR sensors have demonstrated the capacity to identify behavioral deviations, risk events, and functional changes before they become clinically apparent.

Despite the extensive use of PIR sensors for monitoring cognitive and functional status, relatively few studies have examined whether aggregated mobility metrics derived from PIR sensors, such as movement frequency and Temporal Mobility Variability (TMV), are associated with subjective health-related quality of life (HRQoL). Prior research using PIR sensors has focused predominantly on detecting cognitive decline, behavioral signatures associated with specific diseases, or functional impairment, with limited attention to perceived well-being and quality of life as primary outcomes [23,24,25,26]. Moreover, few studies have integrated mobility features derived from PIR sensors with supervised machine learning approaches to discriminate levels of HRQoL.

By explicitly targeting HRQoL as the outcome of interest, the present study complements existing work and extends the application of ambient sensing beyond disease oriented monitoring toward a broader assessment of perceived well-being and functional health in aging populations. Specifically, this study evaluates whether indoor mobility features derived from PIR sensors and collected over a three-month period can distinguish between high and low HRQoL groups among older adults living alone, thereby addressing an important gap in the current literature.

### 2.1. Mobility Metrics

Indoor mobility patterns were quantified using a set of complementary metrics extracted from PIR sensor activations. These measures capture both the amount of movement within the home and the temporal structure of daily activities. In this study, we use the term Temporal Mobility Variability (TMV) to describe the dispersion in the timing of PIR-detected mobility events. TMV reflects variability in movement timing and should not be interpreted as postural or gait stability in the clinical sense.

**Mobility Frequency** was defined as the total number of PIR activations detected for each participant. Because the duration of valid monitoring varied slightly between individuals, raw activation counts were normalized by the number of days with complete sensor data, yielding an average daily mobility frequency. Higher frequency values reflect greater indoor activity levels and may indicate higher functional capacity and independence.

**Temporal Mobility Variability (TMV)** represented the temporal dispersion of movement events and should not be interpreted as postural or gait stability. For each participant, timestamps of valid movement detections were ordered chronologically, and inter-event intervals were computed. Extremely short intervals (<1 s), interpreted as sensor artifacts, were removed. The standard deviation (SD) of all remaining intervals was used as the TMV metric, with larger SD values reflecting more heterogeneous timing of movement and smaller values indicating more regular activity rhythms. As with frequency, TMV was computed after pooling inter-event intervals across all rooms to obtain a global representation of temporal mobility patterns.

In addition to these two primary metrics, several **derived mobility features** were generated to enhance the interpretability and discriminative power of the mobility signal:

- **Log-transformed Frequency** and **Log-transformed TMV**: Applied to reduce skewness and approximate normality in the distribution of mobility measures.
- **TMV-to-Frequency Ratio**: Defined as the participant’s global TMV divided by their normalized frequency, representing the temporal dispersion of movement relative to overall activity volume.
- **Mobility Intensity Index**: A composite indicator calculated as normalized frequency multiplied by the inverse of TMV, capturing the interplay between activity quantity and temporal regularity.

Together, these metrics provide a multidimensional characterization of indoor mobility. Mobility frequency offers a direct measure of daily activity levels, whereas Temporal Mobility Variability captures the temporal regularity or dispersion with which movements occur. The engineered features further contextualize these patterns by modeling scale adjusted variability and combined activity and regularity dynamics. This expanded feature set enables a more comprehensive analysis of behavioral functioning and supports downstream classification tasks using machine learning models.

### 2.2. Importance and Applications

Monitoring mobility frequency and Temporal Mobility Variability (TMV) within the home environment provides valuable insights into the functional status and well-being of older adults. This approach has several key applications:

**Fall Risk Assessment:** Regular tracking of mobility patterns can help identify individuals at higher risk of falls. Changes in TMV and movement frequency may reflect alterations in daily activity organization that have been associated with functional decline and increased fall risk in older adults [28].

**Health Monitoring:** Continuous assessment of in-home mobility can provide healthcare professionals with valuable data to evaluate the health status of older adults, enabling timely medical interventions [29].

**Improving Quality of Life:** Understanding mobility patterns allows for the customization of living environments to better suit the needs of older adults, promoting independence and enhancing their quality of life [30,31,32].

## 3. Quality of Life and the EQ-5D Questionnaire

The EQ-5D questionnaire is a standardized instrument developed by the EuroQol Group to measure health-related quality of life (HRQoL) across different populations and clinical conditions [33,34]. In this study, HRQoL was assessed using the EQ-5D-3L version, which evaluates five dimensions of health status: mobility, self-care, usual activities, pain/discomfort, and anxiety/depression.

### 3.1. Components of the EQ-5D-3L

The EQ-5D-3L consists of two main parts: a descriptive health profile and a health evaluation using a Visual Analog Scale (VAS). The descriptive component assesses five dimensions of health status:

- **Mobility**: Assesses the individual’s ability to move and walk around without assistance.
- **Self-Care**: Measures the individual’s ability to perform basic personal care activities, such as washing and dressing.
- **Usual Activities**: Evaluates the individual’s ability to carry out daily activities, such as working, studying, performing household tasks, and engaging in leisure activities.
- **Pain/Discomfort**: Measures the presence and intensity of pain or discomfort experienced by the individual.
- **Anxiety/Depression**: Assesses the level of anxiety or depression experienced by the individual.

In the EQ-5D-3L version, each dimension is rated using three severity levels: no problems, some problems, and extreme problems. The responses across the five dimensions are combined to create a five-digit health profile that can be converted into a single EQ-5D index value using an appropriate value set [34,35]. This approach allows health status to be summarized quantitatively, facilitating comparisons across individuals, groups, and study populations.

### 3.2. EQ-5D Health Index

The EQ-5D health index is derived from the combination of severity levels reported across the five dimensions. The resulting score is commonly interpreted on a scale in which 1 represents full health and 0 represents a health state equivalent to death. In some cases, health states perceived as worse than death may receive negative values [35]. The EQ-5D is widely used in clinical research and healthcare practice to evaluate interventions, compare treatments, and monitor changes in health status over time. Its simplicity, interpretability, and extensive validation make it a valuable tool for assessing HRQoL in population-based and clinical studies [33,34].

In the context of the present study, the EQ-5D health index served as the main outcome measure for evaluating the relationship between in-home mobility patterns and perceived quality of life in older adults. By integrating PIR-based metrics such as movement frequency, Temporal Mobility Variability (TMV), and derived mobility features, this study combines objective behavioral information collected in the home environment with self-reported health perception. Participants were subsequently grouped according to their EQ-5D index to support the descriptive and supervised classification analyses.

## 4. Methods

### 4.1. Participants

A total of 40 community-dwelling older adults participated in the study. All participants lived alone and independently in private residences in the Valparaíso region of Chile. Descriptive characteristics of the sample, including age distribution, sex, monitoring duration, and EQ-5D scores, are presented in Table 1. Recruitment was conducted through community health centers and local senior organizations using informational sessions, printed notices, and invitations facilitated by healthcare professionals.

Eligibility criteria included living alone, being at least 65 years of age, having adequate cognitive and communicative capacity to understand the study procedures, and providing written informed consent. Older adults with stable and well-controlled chronic conditions, such as hypertension or diabetes, were included if these conditions did not interfere with daily functioning.

Individuals were excluded if they presented moderate or severe cognitive impairment that could compromise adherence to study procedures, had medical or mobility limitations incompatible with safe independent living, or lived in household environments that did not allow proper installation of the sensing equipment. The study protocol was approved by the Scientific Ethics Evaluation Committee of Universidad Andrés Bello, Chile, under approval number 032/2023.

### 4.2. Home Monitoring Setup

**Figure 1:**
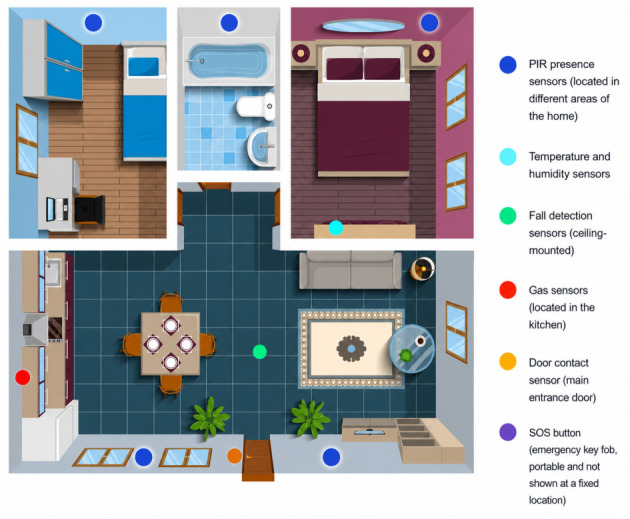
Location of sensors inside homes.

Figure 1 illustrates a typical home layout from the study, consisting of two bedrooms, a combined living and dining area, and a kitchen. Passive Infrared (PIR) sensors were installed in each of these functional areas to continuously monitor indoor mobility patterns.

The home monitoring system used in the present study was based on the Quida platform, an Ambient Assisted Living system previously developed and evaluated in older adults in Chile. Quida integrates a network of non-intrusive sensors installed in the homes of older adults to support continuous monitoring, risk event detection, and assistance while preserving privacy. The platform has been previously described in detail, including its monitoring model, alert protocol, and sensor infrastructure, in a randomized clinical trial evaluating its impact on quality of life among community-dwelling older adults [32]. Related technical developments associated with this monitoring ecosystem have also addressed nocturia monitoring, fall detection, infrared thermal sensing datasets, emergency alert systems, microservice-based implementation, and computational solutions for fall classification [36,37,38,39,40,41].

In some homes, additional devices were installed to enhance safety and contextual monitoring, including a radar sensor for fall detection, a gas sensor in the kitchen, a contact sensor on the main door, and an emergency panic button. These devices were used exclusively for safety, contextual validation, and emergency response, and were not included as analytical predictors in the present study.

For the purposes of this research, only PIR sensor data were utilized, as these devices reliably capture movement-derived activity within the home and allow for unobtrusive monitoring of daily mobility behaviors among older adults living alone.

### 4.3. System Verification and Data Validation

Before the beginning of the monitoring period, the sensor system installed in each home was verified to ensure proper device operation, data communication, and event recording. The verification procedure included activation tests for each Passive Infrared (PIR) motion sensor, confirmation of timestamp synchronization, inspection of data transmission logs, and validation that events detected by sensors were correctly associated with the corresponding area of the home.

The monitoring platform included PIR sensors installed in the main functional areas of the home, as well as additional devices such as a temperature and humidity sensor, a fall detection sensor, a gas sensor, a contact sensor on the main entrance door, and an SOS device. These additional sensors were part of a broader safety and contextual monitoring infrastructure. However, for the purposes of the present analysis, only movement events derived from PIR sensors were used to compute indoor mobility features. Data from the other devices were not used as predictive variables in the machine learning models.

During the monitoring period, data quality was assessed through periodic inspection of activation patterns and communication logs. Implausible activation sequences, such as repeated activations within extremely short intervals, prolonged continuous activation, or extended periods of silence incompatible with known occupancy conditions, were flagged for review. Intervals between consecutive events shorter than 1 s were excluded as possible artifacts caused by sensor reactivation or environmental noise. Prolonged periods without PIR activity were examined together with auxiliary information from the entrance door contact sensor and the SOS device to distinguish between true inactivity, periods outside the home, and possible communication failures.

Intervals corresponding to periods outside the home were excluded from the computation of indoor mobility metrics to ensure that the derived features reflected only behavior within the home. After artifact filtering and exclusion of validated absence periods, clean PIR activation records were used to compute mobility frequency, Temporal Mobility Variability (TMV), and the derived features used in the classification analysis.

To support transparency and reproducibility, Supplementary Figure S1 provides visual documentation of the devices used in the monitoring platform, Supplementary Table S1 summarizes the technical role of each sensor, and Supplementary File S1 presents an anonymized example of the data structure used for feature extraction.

### 4.4. Quality of Life Grouping

Participants were classified into two groups according to their EQ-5D index. A threshold of 0.6 was used to distinguish between lower and higher levels of health-related quality of life. Participants with an EQ-5D score of 0.6 or higher were assigned to the high HRQoL group, whereas those with scores below 0.6 were assigned to the low HRQoL group.

The threshold of 0.6 was selected as a pragmatic cutoff for this exploratory analysis based on the observed distribution of EQ-5D scores within the study sample and the need to obtain groups of sufficient size for supervised classification. This cutoff was not intended to represent a universal clinical threshold. Rather, it was used to provide an interpretable grouping strategy that allowed the evaluation of whether PIR-derived indoor mobility features could discriminate between participants with lower and higher perceived health status.

### 4.5. Filtering of Sensor Artifacts

Raw PIR activation logs may contain spurious or non-physiological events resulting from sensor noise, rapid reactivation, or environmental fluctuations. To ensure the reliability of the mobility metrics, a preprocessing pipeline was implemented to filter these artifacts before computing inter-event intervals and derived mobility features.

First, all PIR activations were sorted chronologically for each participant. Consecutive events occurring within extremely short time windows were treated as non-valid. Specifically, inter-event intervals shorter than 1 s were removed, as these rapid activations do not represent meaningful human movement and may instead reflect sensor reactivation or thermal noise. Second, prolonged periods of inactivity were evaluated to distinguish physiological inactivity, such as nighttime sleep, from data transmission gaps or periods outside the home. Intervals exceeding 3 h were flagged and cross-checked against monitoring logs. Only intervals attributable to sensor malfunction, communication lapses, or validated absence from the home were removed, whereas inactivity related to the participant’s routine was retained.

Third, malfunction events associated with specific sensors, such as continuous firing without movement or prolonged silence despite known occupancy, were identified by inspecting temporal activation patterns. Affected time blocks were excluded from feature computation for the corresponding participant to avoid inflating variance measures. After artifact filtering, clean inter-event intervals were used to derive Temporal Mobility Variability (TMV) and additional engineered features. This preprocessing ensured that variability metrics reflected behavioral patterns rather than sensor-induced noise.

### 4.6. Handling Periods Outside the Home

Periods during which participants were outside the home were identified using auxiliary sensing information available in the monitoring system. A contact sensor installed on the main entrance door was used to detect entry and exit events, providing contextual information about transitions between presence and absence in the home. In addition, participants carried an emergency key fob equipped with GPS, an accelerometer, and a fall detection module, which supported the identification of periods spent outside the home.

Information from the door contact sensor and the emergency key fob was used exclusively to identify and validate intervals outside the home and to distinguish true absence from prolonged inactivity due to sensor malfunction or nighttime rest. All intervals corresponding to periods outside the home were excluded from the computation of indoor mobility metrics to ensure that derived features reflected behavior inside the home only. Data from the emergency key fob and the door sensor were not included as analytical predictors in the present study.

### 4.7. Mobility Metrics and Feature Engineering

Indoor mobility patterns were operationalized using a set of primary and derived metrics extracted from PIR sensor activations. These metrics were designed to capture both the amount of movement occurring within the home and the temporal structure with which daily activities were performed.

**Primary Mobility Metrics.** Two fundamental indicators were computed for each participant:

- **Mobility Frequency**: the total number of PIR activations recorded during the monitoring period. Because the number of valid monitoring days differed slightly across participants, raw activation counts were normalized by dividing by the number of days with complete sensor data, yielding a mobility frequency adjusted by monitoring duration.
- **Temporal Mobility Variability (TMV)**: the temporal dispersion of movement, defined as the standard deviation (SD) of inter-event intervals. Timestamps from all PIR sensors were ordered chronologically, and the time difference between consecutive activations was computed. Intervals shorter than 1 s, interpreted as sensor artifacts, were removed prior to computing the SD. This global TMV measure reflects variability in the rhythm and timing of daily movement patterns and should not be interpreted as postural or gait stability.

**Derived Mobility Features.** To improve the discrimination capacity of the mobility signal and account for skewed distributions, several engineered features were generated:

- **Log-transformed Frequency and Log-transformed TMV**: Applied to reduce skewness and approximate normality in the distribution of mobility measures.
- **TMV-to-Frequency Ratio**: Defined as the participant’s global TMV divided by their normalized frequency, representing the temporal dispersion of movement relative to overall activity volume.
- **Mobility Intensity Index**: A composite indicator calculated as normalized frequency multiplied by the inverse of TMV, capturing the interplay between activity quantity and temporal regularity.

All metrics were standardized through transformation to standard scores prior to model training to ensure equal scaling across features. The resulting feature set provides a multidimensional representation of indoor mobility patterns that incorporates activity volume, temporal organization, and variability adjusted dynamical properties of movement. These engineered features were used as predictors in subsequent machine learning analyses. All metrics were computed after artifact filtering and exclusion of validated periods outside the home, ensuring that the derived features reflected indoor mobility behavior only.

### 4.8. Unit of Analysis for Classification

For the supervised classification analysis, the unit of analysis was the participant. All PIR activations recorded during the monitoring period were aggregated at the participant level to generate one mobility feature vector per individual. Therefore, the final machine learning dataset contained one row per participant, with the corresponding mobility-derived features and a single HRQoL class label derived from the participant’s EQ-5D index. No PIR event-level records, room-level records, or daily observations were treated as independent classification instances.

Because each participant contributed only one aggregated feature vector to the classification dataset, the same participant could not appear simultaneously in both training and test folds during cross-validation. This participant-level aggregation prevented within-subject information leakage and ensured that model performance reflected discrimination between individuals rather than repeated observations from the same individual.

### 4.9. Class Balance Considerations

The original distribution of health-related quality of life (HRQoL) categories was imbalanced, with a higher proportion of participants classified in the high HRQoL group than in the low HRQoL group (27 versus 13 participants). Class imbalance may bias model training by favoring the majority class and limiting the classifier’s ability to learn discriminative patterns associated with the underrepresented group.

To mitigate this issue, class balance was addressed using random oversampling of the minority class. Random oversampling was implemented using the RandomOverSampler algorithm within an *imbalanced-learn* pipeline, thereby integrating the resampling procedure directly into the machine learning workflow. This approach increases the representation of the minority class by duplicating existing training samples without modifying the original feature space.

Importantly, oversampling was performed exclusively within the training partitions of the nested stratified five-fold cross-validation procedure. At each iteration, the minority class was balanced only in the corresponding training data used for model development and hyperparameter optimization. The associated outer test fold retained the original participant distribution and was never oversampled.

By embedding the oversampling procedure within the training pipeline, no duplicated observations were introduced into the evaluation data, thereby preventing information leakage during performance estimation. Consequently, all reported performance metrics were computed exclusively from the original, non-oversampled outer test folds, providing an unbiased estimate of classifier performance under the original class distribution.

In addition to the fold-wise performance metrics reported as mean ± standard deviation across the five outer folds, class-specific precision, recall, and F1-score were calculated from aggregated out-of-fold (OOF) predictions. During the outer cross-validation procedure, each participant was included in an outer test fold exactly once. Predictions from the five outer test folds were then aggregated to generate class-specific performance estimates for the complete cohort while preserving the original class distribution.

### 4.10. Machine Learning Models

Three supervised machine learning algorithms were evaluated to classify participants according to their health-related quality of life (HRQoL) category using passive infrared (PIR)-derived mobility features: Support Vector Machine (SVM) with a radial basis function (RBF) kernel, Random Forest (RF), and K-Nearest Neighbors (KNN). These algorithms were selected because they represent complementary classification approaches and have been widely applied to health-related classification problems involving relatively small datasets.

Model development and evaluation were performed using a nested stratified five-fold cross-validation framework. The outer cross-validation loop was used exclusively to estimate model performance on unseen participants, whereas the inner cross-validation loop was used to optimize model hyperparameters using GridSearchCV. This nested evaluation strategy reduces optimistic bias by ensuring that model selection and performance estimation are conducted on independent data.

To address class imbalance, RandomOverSampler was incorporated into an *imbalanced-learn* pipeline together with the preprocessing steps and the corresponding classifier. Oversampling was applied exclusively to the training partitions of each cross-validation iteration, while the outer test folds retained the original participant distribution. This strategy prevented information leakage and ensured unbiased performance estimation.

Hyperparameter optimization was performed independently for each classifier. For the SVM model, the regularization parameter (*C*) and kernel coefficient (γ) were optimized. For the Random Forest model, the number of trees and maximum tree depth were optimized. For the K-Nearest Neighbors model, the number of neighbors and the weighting strategy were optimized.

Model performance was evaluated using Accuracy, Precision, Recall, F1-score, and the Area Under the Receiver Operating Characteristic Curve (AUC). Performance metrics were computed exclusively from the original, non-oversampled outer test folds and are reported as the mean and standard deviation across the five outer folds.

For ROC analysis, the SVM classifier used the decision function to generate continuous prediction scores, whereas Random Forest and K-Nearest Neighbors used class probability estimates obtained from *predict_proba()*. Mean ROC curves and AUC values were subsequently calculated across the outer cross-validation folds.

## 5. Results

### 5.1. Descriptive Analysis of Mobility Frequency and HRQoL

Before evaluating the supervised machine learning models, frequency-based indoor mobility features were descriptively compared to characterize the mobility patterns included in the classification analysis. Participants were classified into two groups using the EQ-5D threshold defined in this study: low HRQoL for EQ-5D values below 0.6 and high HRQoL for EQ-5D values equal to or greater than 0.6.

**Figure 2:**
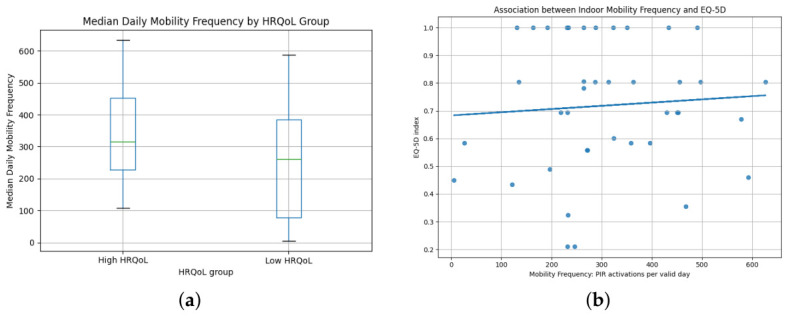
Indoor mobility frequency according to health-related quality of life. (**a**) Distribution of median daily PIR activations in participants with low and high HRQoL, classified using an EQ-5D threshold of 0.6. (**b**) Association between normalized indoor mobility frequency and the EQ-5D index.

As shown in Figure 2, participants in the high HRQoL group presented higher median daily mobility frequency than those in the low HRQoL group. The median daily frequency was 316.0 PIR activations per day in the high HRQoL group and 261.0 PIR activations per day in the low HRQoL group. This difference showed a small effect size according to Cliff’s delta (δ=0.282), although it did not reach statistical significance in the Mann–Whitney U test (p=0.1571).

A similar pattern was observed for mobility frequency normalized by the number of valid monitoring days. For this metric, the high HRQoL group showed a median of 313.6 PIR activations per valid day, whereas the low HRQoL group showed a median of 246.0 PIR activations per valid day. The effect size was also small (Cliff’s delta, δ=0.248), and the between-group comparison did not reach statistical significance (p=0.2144). The association between mobility frequency and the continuous EQ-5D index was weak and non-significant, with Spearman’s ρ=0.042 and p=0.7972.

Overall, both frequency-based metrics showed higher median values in the high HRQoL group. Although these univariate differences were not statistically significant, they provide an interpretable descriptive basis for the subsequent machine learning analysis, in which mobility frequency was evaluated together with temporal and derived mobility features for HRQoL classification.

### 5.2. Model Performance Analysis

**Figure 3:**
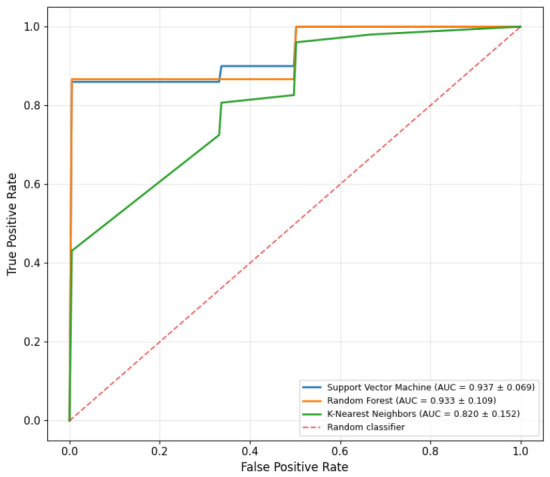
Receiver operating characteristic (ROC) curves of the evaluated machine learning models obtained using nested stratified five-fold cross-validation. The curves represent the mean classification performance across the five outer cross-validation folds. For the Support Vector Machine classifier, ROC analysis was computed using the decision function, whereas Random Forest and K-Nearest Neighbors used predicted class probabilities. The legend reports the mean area under the curve (AUC) and standard deviation across the outer folds.

The predictive performance of the three supervised machine learning models evaluated under the nested stratified five-fold cross-validation framework is summarized in Table 2 and Figure 3. Performance metrics are reported as the mean and standard deviation across the five outer cross-validation folds. As shown in Table 2, the Support Vector Machine (SVM) achieved the best overall performance across the evaluated metrics, whereas Random Forest obtained a comparable AUC despite slightly lower accuracy and F1-score. In contrast, K-Nearest Neighbors (KNN) showed lower overall predictive performance and greater variability across the outer folds.

Class-specific performance obtained from aggregated out-of-fold (OOF) predictions is presented in Table 3. Each participant contributed a single OOF prediction, thereby preserving the original class distribution. The SVM provided the most balanced performance across both HRQoL groups. Random Forest achieved higher performance for the high-HRQoL group but showed lower recall for the low-HRQoL group. Conversely, KNN achieved the highest recall for the low-HRQoL group, although this was accompanied by lower overall accuracy, F1-score, and AUC.

Overall, the SVM achieved the best overall predictive performance, whereas the complementary OOF analysis highlighted differences in class-specific classifier behavior that were not apparent from the fold-wise metrics alone.

## 6. Discussion

The present findings indicate that indoor mobility patterns derived from passive infrared (PIR) sensors contain complementary behavioral information associated with health-related quality of life (HRQoL) in community-dwelling older adults. Although the descriptive analysis did not reveal statistically significant differences for individual mobility metrics, participants with higher HRQoL consistently exhibited higher median values for frequency-based mobility measures. While these univariate differences alone were insufficient to discriminate between HRQoL groups, their direction was consistent with the hypothesis that greater in-home activity is associated with better perceived health status.

From a behavioral perspective, older adults who maintain more frequent movement throughout the day are more likely to preserve functional capacity, independence in activities of daily living, and engagement with their home environment. Indoor mobility therefore reflects more than physical capability alone, encompassing behavioral aspects such as daily routine, motivation, and participation in meaningful activities. Consequently, PIR-derived mobility metrics provide interpretable indicators of everyday functioning that may complement conventional assessments of health-related quality of life.

These findings also support the concept that HRQoL cannot be adequately characterized by isolated mobility indicators. Instead, complementary behavioral features describing activity volume, temporal organization, and daily mobility patterns appear to provide a richer representation of functional status. This observation is consistent with the multidimensional nature of HRQoL, where physical, functional, behavioral, and environmental factors interact to influence perceived health status. Accordingly, the multivariate machine learning models were able to identify relationships that were not evident through conventional univariate statistical analyses.

The superior overall performance achieved by the Support Vector Machine suggests that nonlinear decision boundaries may better capture the complex relationships among indoor mobility characteristics and perceived HRQoL in this dataset. Random Forest achieved comparable discriminative ability, particularly in terms of AUC, further supporting the presence of nonlinear interactions among PIR-derived mobility features. Together, these findings reinforce the value of multivariate machine learning approaches for passive sensing applications, where clinically relevant behavioral patterns are likely to emerge from combinations of complementary variables rather than from individual mobility measures.

Importantly, the complementary aggregated out-of-fold (OOF) analysis provided participant-level class-specific performance estimates while preserving the original class distribution. This analysis revealed differences in classifier behavior that were not fully apparent from the fold-wise performance metrics alone. Although the Support Vector Machine achieved the most balanced performance across both HRQoL groups, Random Forest showed reduced sensitivity for identifying participants with low HRQoL, whereas K-Nearest Neighbors achieved higher recall for the minority class at the expense of lower overall predictive performance. These findings illustrate the trade-offs between overall predictive performance and minority-class sensitivity, highlighting the importance of reporting both fold-wise performance metrics and complementary class-specific analyses when evaluating machine learning models on imbalanced clinical datasets.

The absence of statistically significant univariate associations further indicates that no individual mobility feature should be interpreted as an independent marker of HRQoL in this cohort. Rather, the predictive performance achieved by the multivariate models suggests that complementary information distributed across multiple mobility indicators contributes to the discrimination between HRQoL groups. This finding reinforces the potential of multivariate machine learning approaches for passive sensing applications, where behavioral outcomes are typically influenced by multiple interacting physiological, functional, and environmental factors.

From a clinical and public health perspective, these findings suggest that unobtrusive indoor mobility monitoring may provide clinically relevant behavioral information capable of complementing traditional patient-reported outcome measures. Passive sensing technologies offer the possibility of continuous, ecologically valid assessment without increasing participant burden, making them particularly attractive for long-term monitoring of older adults living independently. Although the proposed models should not be considered clinically deployable at this stage, the results demonstrate the feasibility of using simple, low-cost, and privacy-preserving ambient sensing technologies to investigate HRQoL under real-world living conditions.

An additional strength of the proposed framework is the interpretability of the extracted mobility features. Metrics such as mobility frequency and temporal mobility indicators can be directly related to observable daily behaviors, facilitating clinical interpretation, communication with caregivers, and the design of personalized interventions. Unlike many high-dimensional digital biomarkers, these measures retain clear behavioral meaning while remaining compatible with machine learning models capable of capturing complex relationships among multiple variables. Nevertheless, these metrics should be interpreted as complementary indicators rather than standalone diagnostic markers.

Taken together, these findings support the feasibility of unobtrusive indoor mobility monitoring as a source of clinically meaningful behavioral information for investigating HRQoL in community-dwelling older adults. Future studies including larger, independent, and more diverse cohorts will be essential to confirm the generalizability of these findings and to further evaluate the potential of PIR-derived mobility features as digital biomarkers for healthy aging.

## 7. Conclusions

The integration of PIR sensors with machine learning algorithms represents a promising strategy for assessing indoor mobility and exploring its relationship with health-related quality of life (HRQoL) in older adults. Although the univariate analysis did not identify statistically significant differences between HRQoL groups, the multivariate analysis demonstrated that complementary mobility features extracted from PIR sensors contain meaningful information for discriminating perceived health status.

Using a nested stratified five-fold cross-validation framework with RandomOverSampler applied exclusively within the training folds, the evaluated machine learning models achieved encouraging predictive performance. Among them, the Support Vector Machine provided the best overall performance, whereas Random Forest achieved comparable discriminative ability. The complementary class-specific analysis based on aggregated out-of-fold predictions further highlighted differences in classifier sensitivity between HRQoL groups, emphasizing the importance of evaluating both overall and class-specific performance when analyzing imbalanced datasets.

From a clinical and public health perspective, these findings support the potential value of unobtrusive mobility monitoring as a complementary approach to traditional self-reported assessments. Passive digital biomarkers derived from PIR sensors may contribute to continuous, ecologically valid observation of daily functioning in the home environment. Nevertheless, the proposed models should be considered proof-of-feasibility rather than clinically deployable predictive tools, given the relatively small study cohort.

Overall, this study demonstrates the feasibility of combining unobtrusive ambient sensing with interpretable machine learning models to investigate HRQoL in community-dwelling older adults. The proposed framework provides a foundation for future digital health applications aimed at supporting healthy aging through continuous, home-based functional assessment. Future studies including larger, independent, and more balanced cohorts are needed to validate these findings and further evaluate the potential of PIR-derived mobility features as digital biomarkers of healthy aging.

## 8. Limitations and Future Work

This study should be interpreted in light of several limitations. First, the sample size was modest, with 40 community-dwelling older adults monitored over a three-month period. Although the longitudinal nature of the sensor data represents a strength of the study, the machine learning dataset was constructed at the participant level, resulting in one aggregated feature vector per individual. Therefore, the number of independent observations for classification was limited. This may have reduced statistical power, increased uncertainty in model performance estimates, and limited the generalizability of the findings.

Second, indoor mobility patterns may be influenced by contextual factors that were not fully captured in the present analysis. These include differences in home architecture, room size, sensor placement, daily routines, lifestyle preferences, social engagement, and psychosocial conditions. Although participants shared similar general living conditions, variability in housing layouts and personal routines may have introduced heterogeneity not directly attributable to health-related quality of life. Future studies should incorporate more detailed contextual information about the home environment and daily activity structure.

Third, the dichotomization of EQ-5D using a threshold of 0.6 should be interpreted as a pragmatic grouping strategy rather than as a clinically validated cutoff. This approach facilitated supervised classification and provided an interpretable comparison between participants with lower and higher perceived health status. However, it necessarily simplified the continuous and multidimensional nature of HRQoL. Future research with larger cohorts should evaluate the robustness of alternative EQ-5D thresholds and consider regression-based approaches using the continuous EQ-5D index.

Fourth, although the classification analysis was performed at the participant level and no participant could appear simultaneously in the training and test partitions, the limited number of observations in the minority HRQoL group remains an important limitation. Random oversampling was applied exclusively within the training partitions of the nested stratified five-fold cross-validation procedure, whereas all performance metrics and class-specific out-of-fold predictions were obtained from the original non-oversampled outer test folds. Although this strategy minimizes information leakage, the limited number of participants may still contribute to variability in class-specific performance estimates, particularly for the minority class. Accordingly, the reported classification results should be interpreted as proof-of-feasibility rather than evidence of clinical readiness.

Fifth, the present analysis used aggregated PIR-derived mobility metrics and did not fully model spatial context or room-specific behavior. While this approach provides a global characterization of indoor mobility, it does not differentiate the functional meaning of activity occurring in different areas of the home, such as bedrooms, kitchens, bathrooms, or living rooms. Incorporating room-specific activity patterns, transitions between functional areas, and time-of-day profiles may improve the characterization of daily functioning in future studies.

Finally, the study did not include external validation in an independent cohort. Future work should validate the proposed framework in larger, independent, and more balanced populations, ideally across different housing contexts, geographic regions, and health profiles. Additional data sources, including clinical assessments, self-reported behavioral measures, contextual environmental information, or physiological indicators, may further improve model interpretability and predictive performance. Longitudinal analyses focused on within-person behavioral changes may also facilitate the early identification of functional decline or deterioration in health-related quality of life.

## Figures and Tables

**Table 1:** Description of the study sample.

	Age		Monitoring Days		EQ-5D	
	Female	Male	Female	Male	Female	Male
Valid	34	6	34	6	34	6
Mean	75.44	78.43	103.71	95.86	0.693	0.842
Std. Deviation	6.43	7.25	22.30	4.95	0.242	0.115

**Table 2:** Performance of the evaluated machine learning models obtained using nested stratified five-fold cross-validation. Performance metrics were computed exclusively from the original non-oversampled outer test folds and are reported as mean ± standard deviation across the five outer folds.

Model	Accuracy	Precision	Recall	F1-Score	AUC
Support Vector Machine (SVM)	0.825±0.143	0.860±0.080	0.900±0.149	0.876±0.102	0.937±0.069
Random Forest	0.775±0.105	0.803±0.052	0.900±0.149	0.842±0.075	0.933±0.109
K-Nearest Neighbors (KNN)	0.750±0.177	0.860±0.142	0.740±0.224	0.787±0.178	0.820±0.152

**Table 3:** Class-specific performance obtained from the aggregated out-of-fold (OOF) predictions across the five outer cross-validation folds. Support corresponds to the original participant distribution (13 low HRQoL and 27 high HRQoL participants), without oversampling of the evaluation data.

Model	HRQoL Group	Precision	Recall	F1-Score	Support
SVM	Low HRQoL	0.750	0.692	0.720	13
SVM	High HRQoL	0.857	0.889	0.873	27
Random Forest	Low HRQoL	0.700	0.538	0.609	13
Random Forest	High HRQoL	0.800	0.889	0.842	27
KNN	Low HRQoL	0.588	0.769	0.667	13
KNN	High HRQoL	0.870	0.741	0.800	27

## Data Availability

Due to ethical and privacy considerations associated with continuous in-home monitoring of older adults, the raw dataset analyzed in this study is not publicly available. However, an anonymized example of the data structure and the feature extraction procedure is provided as Supplementary Material. Additional aggregated or de-identified data may be made available upon reasonable request to the corresponding author, subject to approval by the research team and in accordance with institutional ethical guidelines.

## Supplementary Materials

The following supporting information can be downloaded at: https://www.mdpi.com/article/10.3390/s26154759/s1, Figure S1: Visual documentation of selected sensors included in the Quida monitoring platform; Table S1: Technical role of sensors included in the Quida monitoring platform; File S1: An anonymized example of the sensor log structure used by the Quida monitoring platform.

## Abbreviations

The following abbreviations are used in this manuscript:

OP	Older people
PIR	Passive Infrared
TMV	Temporal Mobility Variability
HRQoL	Health-related quality of life
SVM	Support Vector Machine
RF	Random Forest
KNN	K-Nearest Neighbors
ADL	Activities of Daily Living
IADL	Instrumental Activities of Daily Living
