# Supplementary material for: Indoor Mobility Patterns Measured by PIR Sensors for Classifying Health-Related Quality of Life in Older Adults Using Machine Learning
Source: Sensors (Basel). 2026 Jul 27;26(15):4759. doi: 10.3390/s26154759 (PMC13468884; doi:10.3390/s26154759)
Supplement: Supplementary file 1 [file sensors-26-04759-s001.zip › Supplementary_Figure_S1_Quida_Monitoring_Devices.pdf]

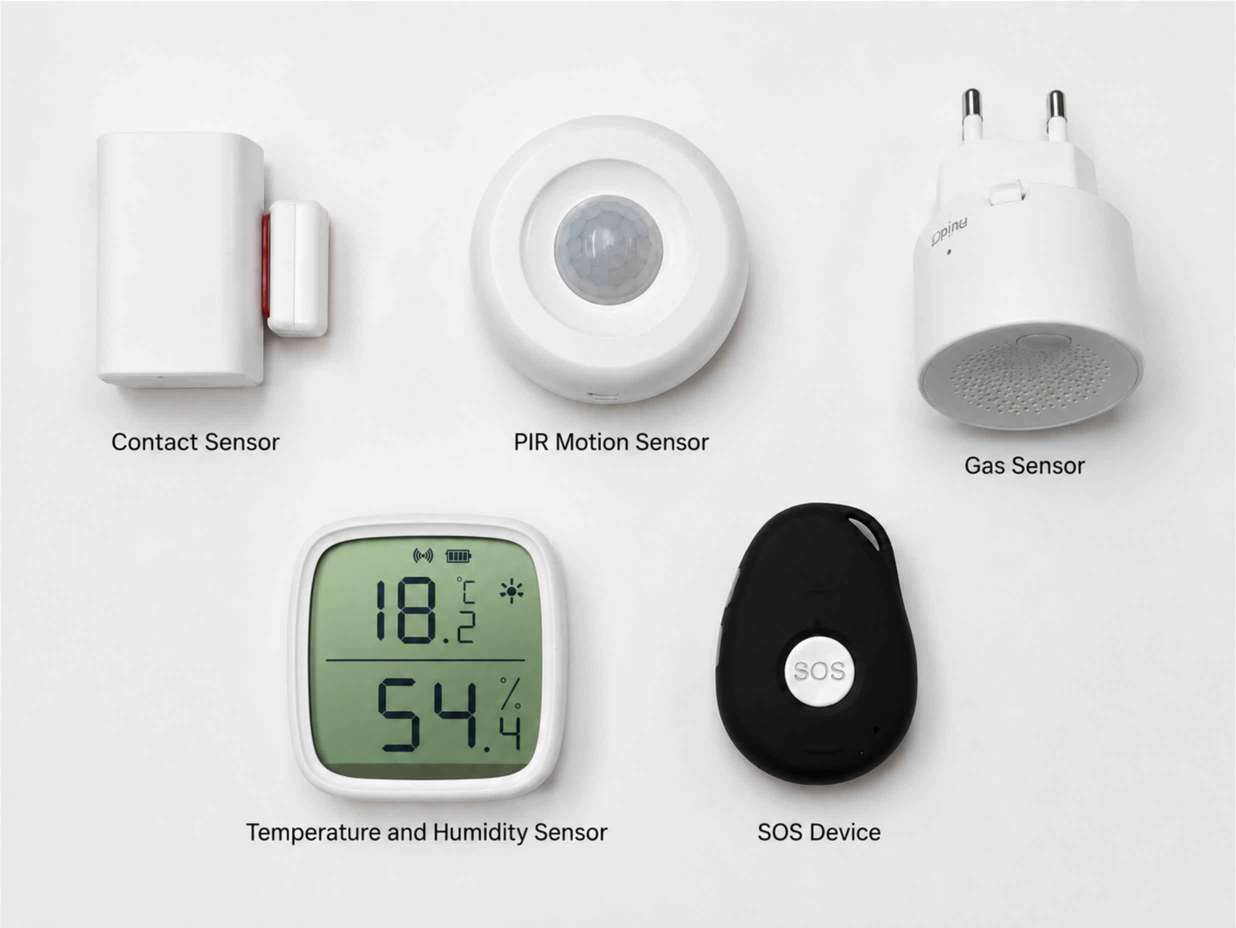

Figure S1: Real photograph of the sensor devices included in the Quida monitoring platform and deployed in the study. The figure shows the contact sensor installed on the main entrance door, PIR motion sensor, gas sensor, temperature and humidity sensor, and SOS emergency key fob. These devices were used for indoor mobility monitoring, safety monitoring, contextual validation, and emergency response. Only PIR motion sensor data were used as analytical predictors in the machine learning models.
