# Supplementary material for: Indoor Mobility Patterns Measured by PIR Sensors for Classifying Health-Related Quality of Life in Older Adults Using Machine Learning
Source: Sensors (Basel). 2026 Jul 27;26(15):4759. doi: 10.3390/s26154759 (PMC13468884; doi:10.3390/s26154759)
Supplement: Supplementary file 1 [file sensors-26-04759-s001.zip › Supplementary_File_S1_Anonymized_Sensor_Log_Structure.pdf]

Supplementary File S1

Anonymized example of the Quida sensor log structure

Table S2: Data fields included in the anonymized sensor log example.

**Field**	**Example value**	**Description**	**Use in this study**
record_id	R0001	Anonymized record identifier	Used only to identify each row in the example file
sensor_code	S005	Anonymized sensor identifier	Used to distinguish events generated by different devices
gateway_code	G001	Anonymized gateway or Home Assistant identifier	Used to represent the local communication unit
home_code	H001	Anonymized home identifier	Used to group records from the same monitored home
room_code	ROOM004	Anonymized room identifier	Used to indicate the home area where the sensor was installed
participant_code	P001	Anonymized participant identifier	Used to link records to one participant without revealing identity
created_at	2024-03-12 18:30:40	Timestamp when the record was created in the system	Used for data management and synchronization checks
measured_at	2024-03-12 11:30:40	Timestamp when the event was measured by the device	Used to order sensor events and compute time intervals
send_measured_at	2024-03-12 11:30:40	Timestamp sent by the device or gateway	Used for verification of data transmission
value	on, off, 21.4, alert	Recorded value of the sensor event	Used according to the measurement type
is_binary	True or False	Indicates whether the recorded value is binary	Used to distinguish binary events from continuous measurements
measurement_type	OCCUPANCY, CONTACT, GAS, TEMPERATURE, HUMIDITY, SOS_EVENT	Type of measurement reported by the sensor	Used to identify PIR events, contextual validation events, safety events, or environmental variables
gps_available	Yes or No	Indicates whether the event may be associated with an external location source	Used only for contextual validation of periods outside the home. GPS coordinates are not reported

Note. This supplementary file provides an anonymized example of the sensor log structure used by the Quida monitoring platform. Participant, home, room, gateway, sensor, and record identifiers were replaced by generic codes. Dates were replaced by fictitious dates while preserving the timestamp format and temporal structure. GPS coordinates are not reported to protect participant privacy. The field *gps_available* only indicates whether an event may be associated with an external location source, such as the SOS emergency key fob. Only PIR occupancy events were used to compute the mobility features included in the machine learning models.

Table S3: Short anonymized example of sensor log records.

Record	Sensor	Room	Value	Measured at	Binary	Type	Use in study
R0001	S005	ROOM004	on	2024-03-12 11:30:40	True	OCCUPANCY	PIR event used to describe indoor mobility
R0002	S006	ROOM005	off	2024-03-12 11:30:50	True	OCCUPANCY	PIR event used to compute movement timing
R0003	S004	ROOM003	on	2024-03-12 11:32:07	True	OCCUPANCY	PIR event used to compute mobility features
R0004	S001	ROOM001	off	2024-03-12 11:37:15	True	CONTACT	Door contact event used for contextual validation
R0005	S007	ROOM003	0	2024-03-12 11:38:25	False	GAS_VALUE	Safety monitoring variable, not used as predictor
R0006	S008	ROOM001	21.4	2024-03-12 11:39:40	False	TEMPERATURE	Environmental context, not used as predictor
R0007	S008	ROOM001	48.2	2024-03-12 11:40:49	False	HUMIDITY	Environmental context, not used as predictor
R0008	SOS001	OUTSIDE	alert	2024-03-12 11:50:00	True	SOS_EVENT	Emergency key fob event used only to support validation of periods outside the home
