# Supplementary material for: Indoor Mobility Patterns Measured by PIR Sensors for Classifying Health-Related Quality of Life in Older Adults Using Machine Learning
Source: Sensors (Basel). 2026 Jul 27;26(15):4759. doi: 10.3390/s26154759 (PMC13468884; doi:10.3390/s26154759)
Supplement: Supplementary file 1 [file sensors-26-04759-s001.zip › Supplementary_Table_S1.pdf]

Table S1: Technical role of sensors included in the Quida monitoring platform.

**Sensor**	**Location or use context**	**Main function**	**Role in this study**	**Predictor**
Main entrance door contact sensor	Main entrance door	Detects door opening and closing through a magnetic contact mechanism	Supports contextual validation of entrance and exit events and helps identify periods outside the home	No
PIR 360 Zigbee motion sensor	Main functional areas of the home, including bedrooms, living or dining areas, and kitchen	Detects indoor movement through passive infrared sensing	Primary source of indoor mobility data used to compute mobility frequency, Temporal Mobility Variability (TMV), and derived features	Yes
Zigbee gas sensor	Kitchen	Detects natural gas and liquefied gas and emits an acoustic alarm when gas concentration exceeds the alert threshold	Part of the safety and emergency monitoring infrastructure	No
Sonoff SNZB-02D Zigbee temperature and humidity sensor	Indoor home environment	Measures temperature and humidity within an enclosed space	Provides contextual environmental information within the Quida platform	No
SOS emergency key fob EV-07b	Carried by the participant, including use outside the home	Sends emergency alerts, supports GPS location, enables phone communication, and detects abrupt vertical movements associated with falls	Supports safety monitoring and validation of periods outside the home. Not used to compute mobility features	No

Note. Only the PIR 360 Zigbee motion sensor was used as an analytical predictor in the machine learning models. The remaining devices were used for safety monitoring, contextual validation, emergency response, or identification of periods outside the home.
